# Adjuvant chemotherapy versus chemoradiation in high‐risk pancreatic adenocarcinoma: A propensity score‐matched analysis

**DOI:** 10.1002/cam4.2491

**Published:** 2019-08-15

**Authors:** Mustafa Raoof, Andrew M. Blakely, Laleh G. Melstrom, Byrne Lee, Susanne G. Warner, Vincent Chung, Gagandeep Singh, Yi‐Jen Chen, Yuman Fong

**Affiliations:** ^1^ Department of Surgery City of Hope National Medical Center Duarte California; ^2^ Department of Medical Oncology City of Hope National Medical Center Duarte California; ^3^ Department of Radiation Oncology City of Hope National Medical Center Duarte California

**Keywords:** adjuvant chemotherapy, adjuvant radiation, margin‐positive, node‐positive, pancreatic cancer

## Abstract

**Purpose:**

The American Society of Clinical Oncology guidelines recommend adjuvant chemoradiation (ACR) for margin‐positive (R1) and/or node‐positive (N+) pancreatic cancers. Our goal was to investigate if there is evidence of superiority of adjuvant chemoradiation (ACR) over adjuvant chemotherapy (AC).

**Methods:**

We utilized data from the National Cancer Database (NCDB) for N+ and/or R1 pancreatic adenocarcinoma patients diagnosed from 2004 to 2012 who underwent ACR or AC. Patients who received neoadjuvant radiation, no adjuvant treatment, or adjuvant radiation alone were excluded. Propensity score nearest‐neighbor 1:1 matching (PSM) was performed between ACR and AC groups based on age, sex, race, insurance, year of diagnosis, comorbidities, tumor site and size, T‐stage, nodal status, margin status, grade, and treatment facility. Primary outcome was overall survival (OS).

**Results:**

A total of 8297 patients were eligible. After PSM, two well‐balanced groups of 3244 patients each were analyzed. ACR resulted in superior OS compared with AC alone (Hazard ratio [HR] 0.83, 95% CI 0.79‐0.87; median OS 22 vs 19 months, *P* < .0001). Subset analyses demonstrated OS benefit of ACR compared with AC in N+, R0 patients (HR: 0.82, 95% CI 0.77‐0.88; Median OS 24 vs 20 months, *P* < .001) as well as N+, R1 patients (HR: 0.77, 95% CI 0.68‐0.87; Median OS 17 vs 15 months, *P* < .001); but not in node‐negative, R1 patients (HR: 1.12, 95% CI 0.84‐1.48; Median OS 18 vs 22 months, *P* = .63).

**Conclusion:**

The addition of radiation to AC was associated with a clinically small but meaningful increase in survival of patients undergoing curative‐intent pancreatic resections. This association was not evident in patients with microscopically positive margins but node‐negative disease and larger studies will be needed.

## Graphical abstract

The American Society of Clinical Oncology guidelines recommend adjuvant chemoradiation for margin‐positive (R1) and/or node‐positive (N+) pancreatic cancers. However, it is unclear if addition of adjuvant radiation to chemotherapy improves overall survival in this high‐risk cohort. In this propensity‐matched analysis of high‐risk pancreatic cancer patients (R1 or N+) from a large clinical oncology database, we evaluate the benefit of adding adjuvant radiation to chemotherapy. We found evidence of superiority of adjuvant chemoradiation over adjuvant radiation alone in N+ patients but not in R1N0 patients.

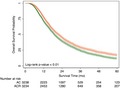

## 1 INTRODUCTION

Pancreatic cancer represents the tenth most common cancer diagnosis and the third most common cause of cancer‐related mortality in the United States, with 55 440 new cases and 44 330 deaths in 2018.^1^ Overall 5‐year survival for all patients is less than 10%.^1^

In a quarter of patients, pancreatic cancer is localized to the pancreas on imaging. Despite potentially curative resection in patients with localized pancreatic cancer, the 5‐year survival rate in these patients is <30%.^1^, ^2^ Almost all patients will have a recurrence with a significant component of locoregional failure (50%‐85%).^2^, ^3^, ^4^ Adjuvant therapy is administered to prevent local recurrence and potentially improve overall survival (OS).

While the role of adjuvant chemotherapy (AC) for pancreatic cancer is well established,^5^, ^6^ the benefit of adding radiation to adjuvant treatment remains unclear. This uncertainty stems from conflicting clinical trial results. For instance, Gastrointestinal Tumor Study Group (GITSG) 9173 was the first randomized trial to demonstrate the benefit of adjuvant 5‐FU‐based chemoradiation compared with observation alone.^7^ In efforts to reproduce these findings, European Organization for Research and Treatment of Cancer (EORTC) conducted a similar trial by randomly assigning 114 patients to observation or 5‐FU‐based chemoradiation after curative‐intent resection.^8^ In contrast to the GITSG trial results, the survival in the two groups was comparable (26% vs 34% for control and treated patients, respectively, *P* = .099). Furthermore, the European Study Group for Pancreatic Cancer trial (ESPAC‐1) demonstrated that while AC was beneficial, addition of radiation therapy was detrimental in patients with pancreatic cancer.^9^ Despite methodological concerns, the ESPAC‐1 trial was influential and partly accounted for a decreasing trend in utilization of adjuvant radiation in the US.^10^, ^11^

At present, adjuvant radiation is used selectively in patients that are thought to be at high risk for locoregional failure (ie, margin and/or node positivity). This is also reflected in professional society guidelines.^12^, ^13^ While there are many retrospective studies that have shown benefit of adjuvant radiation, none of them directly address the utility of radiation for margin‐positive and/or node‐positive pancreatic cancer patients which is the scope of current practice.^10^, ^11^, ^14^, ^15^, ^16^, ^17^

The goal of the present study was to conduct a propensity score‐matched comparison of OS in margin and/or node‐positive patients receiving adjuvant chemotherapy (AC) vs chemoradiotherapy (ACR) using a large national clinical oncology database. Based on prior literature, we hypothesized that ACR is superior to AC in terms of OS.

## 2 METHODS

### 2.1 National Cancer Database patient data

The NCDB, jointly sponsored by the Commission on Cancer (CoC) of the American College of Surgeons and the American Cancer Society, is a nationwide oncology outcomes database based on more than 1400 CoC programs, covering approximately 70% of new cancer cases in the USA.^18^, ^19^ The CoC designates cancer programs based on ability to provide a wide range of oncological services and specialists. CoC‐approved hospitals are larger, perform more operations, and provide more cancer‐related services to patients than non‐CoC hospitals.^19^

The NCDB shared files contain site‐specific de‐identified data on more than 80 variables comprising sociodemographic, tumor, treatment, and follow‐up information. These data are abstracted by certified tumor registrars from medical records, even if the care extends to a non‐CoC facility. The NCDB does not specify the frequency of follow‐up, but sets the standard of 90% at 5 years. Quality is assured by the CoC by means of more than 600 electronic automated checks to maximize internal consistency and minimize missing data. In addition, the CoC also performs routine audits to ensure data quality and completeness.^20^ Institutional review board approval was not required for this study as patient de‐identified data were analyzed.

#### 2.1.1 Patient eligibility and exclusion criteria

Patients diagnosed with pancreatic adenocarcinoma between 2004 and 2012 were identified based on the ICD‐O histology codes (8140, 8480, 8500) and topography code (C25). Patients were excluded if they had clinically metastatic disease at diagnosis or in situ disease. Patients who did not receive adjuvant therapy or received adjuvant radiation alone were excluded. Similarly, patients who were margin and node negative were excluded from the analysis. We also excluded patients who received neoadjuvant therapy.

#### 2.1.2 Patient's data variables and definitions

Variables included age, ethnicity, sex, Charlson‐Deyo score^21^ for co‐morbid conditions, insurance type, hospital type, tumor extent, tumor size, node status, and receipt of adjuvant therapies. AC was defined as any single or combination chemotherapy as part of the planned first course treatment. ACR was defined as systemic chemotherapy in addition to radiation therapy regardless of radiosensitizing chemotherapy. Margin positivity was defined as microscopic residual tumor that cannot be seen by the naked eye (R1). Margin negative resection was defined as no microscopic evidence of tumor at the inked margin according to the assessment of the pathologist (R0). Patients with grossly involved margins or if margins were not accurately assessed were excluded. Staging was based on the AJCC staging manual (seventh edition).^22^ Missing data were reported as separate categories, as they may differ systematically between the two groups.^23^

### 2.2 Propensity score matching

Propensity scores were generated using a multivariable logistic regression model based on selected co‐variables. Initial selection of co‐variables for this model was based on a backward‐stepwise approach (threshold for elimination *P* < .200) and the optimization was theory‐driven. Specifically, all variables associated with the type of adjuvant therapy and thought to be strongly associated with OS were included. Also included were co‐variables that are not associated with type of adjuvant therapy, but are known to be associated with OS as this is known to reduce bias in propensity score matching.^24^ Two well‐balanced groups were created using a greedy matching algorithm (1:1 ratio without replacement) with a caliper radius of 0.001.^25^ Bias reduction was confirmed by Rubin's criteria^26^: Rubin's B (the absolute standardized difference of the means of the linear index of the propensity score in the treated and (matched) non‐treated group) and Rubin's R (the ratio of treated to (matched) non‐treated variances of the propensity score index). Rubin recommends that B be less than 25%, and R between 0.5 and 2, for the samples to be considered sufficiently balanced.

Final adjustment of the model was an iterative process, and was performed by including or excluding variables until the bias reduction was maximized. Based on this final model, the groups were matched on the age, sex, race/ethnicity, insurance, comorbidities, year of diagnosis, hospital type, location and size of the tumor, extent of surgery, pathologic T category, grade, nodal status, and margin status.

### 2.3 Statistical analysis

All analyses were performed on an as‐treated basis. Continuous variables are presented as median (i.qr) and categorical variables as frequencies with percentages. Patient demographics, cancer‐specific, and hospital‐level characteristics were analyzed using Kruskal‐Wallis test for continuous data and the Pearson chi‐square test for categorical data.

Overall survival was calculated from the date of diagnosis until the date of death. Survivors were censored at the date of last contact, whereas those who died were censored at the date of death. Kaplan‐Meier curves were used to depict survival differences between the two groups, and the log‐rank test was used to test these differences for statistical significance. The proportional hazards assumption was confirmed by review of Schoenfeld residuals as well as graphically. Hazard ratios (HRs) are reported with 95% confidence intervals.

A subgroup analysis using Cox proportional hazards model was performed in the propensity score‐matched cohort to determine whether certain subgroups did not benefit from adjuvant chemoradiation.

For all analyses, two‐sided *P* < .050 was considered statistically significant. All analyses were performed using STATA^®^/MP version 14 (StataCorp).

## 3 RESULTS

### 3.1 Patients

**Figure 1:**
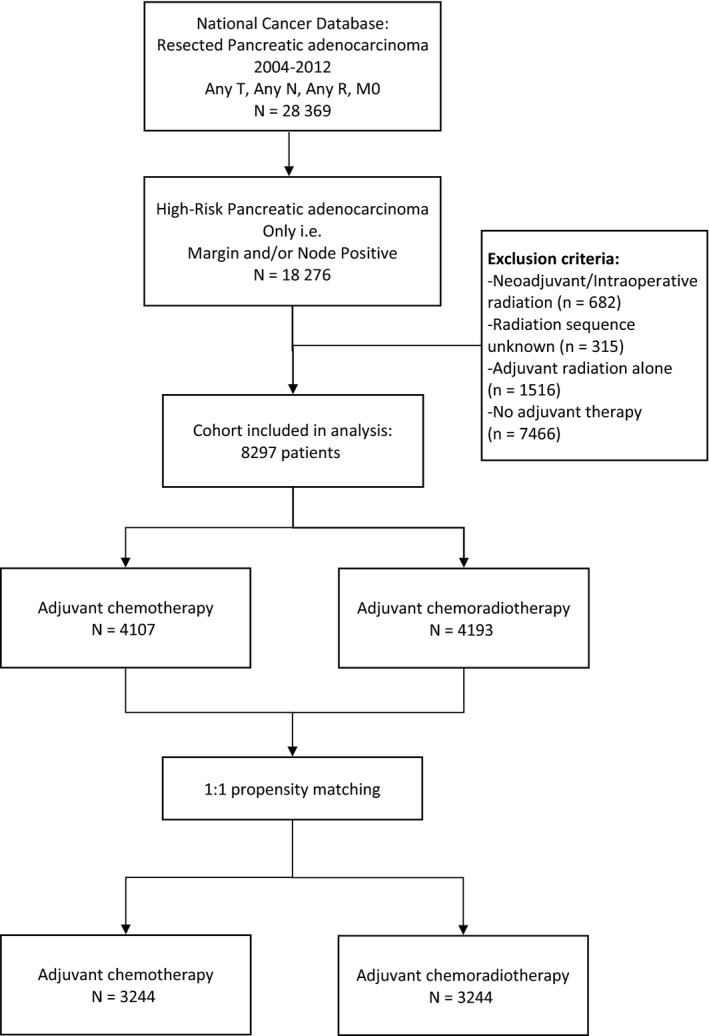
CONSORT Diagram

Overall, 8297 patients were identified from the database, 4104 of whom received AC alone and 4193 of whom received ACR (Figure 1). Analysis of patient, tumor, and procedure information for the two groups showed that patients who underwent ACR were younger, more frequently male, and diagnosed earlier in the study timeframe. ACR patients also had lower T and N stages, higher grade, and more frequent positive resection margin. (Table 1). These differences highlight the selection bias in the use of adjuvant radiation therapy in pancreatic cancer.

**Table 1:** Demographic and clinicopathologic characteristics of patients receiving adjuvant therapy

Characteristic	Original patient cohort			Matched patient cohort		
	Chemotherapy (n = 4104)	Chemoradiation (n = 4193)	*P*‐Value	Chemotherapy (n = 3244)	Chemoradiation (n = 3244)	*P*‐Value
Age (years), n (%)						
<60	1093 (26.6)	1413 (33.7)	**<.001**	946 (29.1)	980 (30.2)	.49
60‐79	2655 (64.7)	2628 (62.7)		2169 (66.9)	2125 (65.5)	
≥80	356 (8.7)	152 (3.6)		129 (4.0)	139 (4.3)	
Sex, n (%)						
Male	2060 (50.2)	2256 (53.8)	**.001**	1713 (52.8)	1691 (52.1)	.58
Female	2044 (49.8)	1937 (46.2)		1531 (47.2)	1553 (47.9)	
Charlson‐Deyo score, n (%)						
0	2627 (64.0)	2731 (65.1)	.51	2084 (64.3)	2096 (64.6)	.94
1	1169 (28.5)	1147 (27.4)		929 (28.6)	922 (28.4)	
2+	308 (7.5)	315 (7.5)		231 (7.1)	226 (7.0)	
Race, n (%)^a^						
Non‐Hispanic White	3418 (83.3)	3433 (81.9)	.37	2711 (83.6)	2701 (83.3)	.91
Black	368 (9.0)	422 (10.1)		312 (9.6)	314 (9.7)	
Hispanic	159 (3.9)	167 (4.0)		127 (3.9)	125 (3.8)	
Other	132 (3.2)	134 (3.2)		94 (2.9)	104 (3.2)	
Insurance, n (%)^a^						
Private	1557 (37.9)	1979 (47.2)	**<.001**	1380 (42.6)	1428 (44.0)	.48
Government	2409 (58.7)	2071 (49.4)		1785 (55.0)	1741 (53.7)	
Uninsured	97 (2.4)	105 (2.5)		79 (2.4)	75 (2.3)	
Year of diagnosis, n (%)						
2004‐2008	1320 (32.2)	1719 (41.0)	**<.001**	1159 (35.7)	1154 (35.6)	.90
2009‐2013	2784 (67.8)	2474 (59.0)		2085 (64.3)	2090 (64.4)	
Facility type, n (%)^a^						
Academic	2736 (66.7)	2462 (58.7)	**<.001**	2063 (63.6)	2068 (63.8)	.90
Non‐academic	1338 (32.6)	1687 (40.2)		1181 (36.4)	1176 (36.2)	
Tumor site, n (%)						
Head	3091 (75.3)	3263 (77.8)	**.040**	2502 (77.1)	2479 (76.4)	.91
Body	216 (5.2)	207 (4.9)		162 (5.0)	163 (5.0)	
Tail	425 (10.4)	369 (8.8)		309 (9.5)	317 (9.8)	
Unspecified/Overlapping	372 (9.1)	354 (8.5)		271 (8.4)	285 (8.8)	
Tumor Size, mm Median, [IQR]	33 [35‐44]	32 [25‐42]	.35	34 [25‐44]	32 [25‐42]	.27
AJCC pT‐stage, n (%)						
T1	139 (3.4)	145 (3.5)	**.019**	113 (3.5)	111 (3.4)	.83
T2	510 (12.4)	549 (13.1)		422 (13.0)	441 (13.6)	
T3	3374 (82.2)	3372 (80.4)		2641 (81.4)	2623 (80.9)	
T4	76 (1.8)	123 (2.9)		67 (2.1)	66 (2.0)	
Tx	5 (0.1)	4 (0.1)		1 (<0.1)	3 (0.1)	
AJCC pN‐stage, n (%)						
N0	153 (3.7)	295 (7.0)	**<.001**	139 (4.3)	149 (4.6)	.55
N1	3951 (96.3)	3898 (93.0)		3105 (95.7)	3095 (95.4)	
Resection margin, n (%)						
R0	3302 (80.5)	2975 (71.0)	**<.001**	2526 (77.9)	2508 (77.3)	.59
R1	802 (19.5)	1218 (29.0)		718 (22.1)	736 (22.7)	
Grade, n (%)						
Low	298 (7.3)	378 (9.0)	**.007**	252 (7.8)	258 (7.9)	.90
Intermediate	2042 (49.7)	2089 (49.8)		1636 (50.4)	1606 (49.5)	
High	1628 (39.7)	1567 (37.4)		1247 (38.4)	1267 (39.1)	
Unknown	136 (3.3)	159 (3.8)		109 (3.4)	113 (3.5)	
Type of surgery, n (%)						
Pancreaticoduodenectomy	3003 (73.1)	3158 (75.3)	.15	2431 (74.9)	2402 (74.0)	.86
Total pancreatectomy	537 (13.1)	499 (11.9)		394 (12.2)	409 (12.6)	
Partial pancreatectomy	525 (12.8)	494 (11.8)		387 (11.9)	402 (12.4)	
Unspecified	39 (1.0)	42 (1.0)		32 (1.0)	31 (1.0)	

Abbreviations: AJCC, American Joint Commission on Cancer; IQR, interquartile range.

a Information was not complete for ≤1% of patient population in the unmatched cohort. Bolded values depict *P* < .05.

### 3.2 Propensity score matching results

To address this bias, propensity score matching was performed on all relevant covariates. Kernel density distribution plots for the two treatment groups are shown in Figure S1. Groups in the unmatched cohort were significantly imbalanced (Rubin's B = 47.8%, Rubin's R = 0.96; *P* < .001, likelihood ratio test of joint insignificance of all regressors). A propensity‐matching algorithm resulted in two well‐balanced groups of 3244 patients each, with a bias reduction (Rubin's B = 15.8%, Rubin's R = 1.26; *P* = .248).

After matching, radiation treatment characteristics in the ACR group were analysed. In most patients, (68%) radiation was given early during adjuvant therapy (ie, before chemotherapy or within 60 days of the start of chemotherapy). Total radiation dose was 5040 cGy or higher in 54%, 4500 cGy to <5040 cGy in 37%, and <4500 cGy in 9% of patients. Radiation boost was given in 817/3244 patients (25%). Intensity‐modulated radiation therapy (IMRT) was used in 920/3244 (28%) of patients. IMRT use is likely underestimated because in 803/3244 (25%) patients, the type of external beam radiation was not specified.

### 3.3 Overall survival in ACR vs AC after propensity score matching analysis

**Figure 2:**
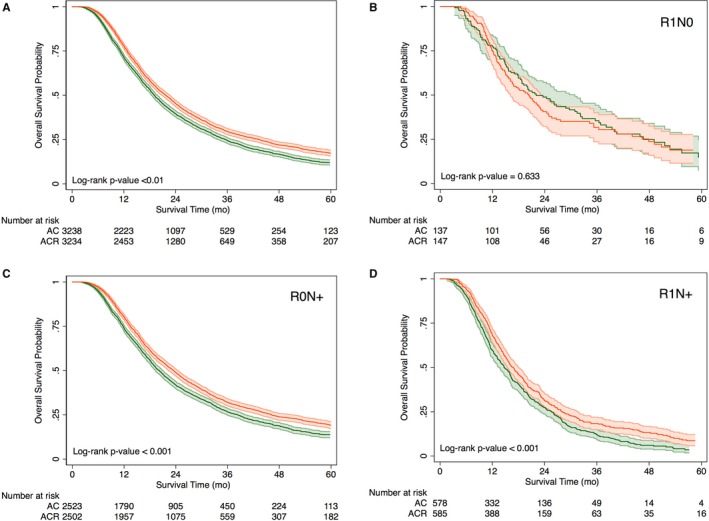
Kaplan‐Meier overall survival estimates, with 95% confidence intervals, of patients undergoing AC (green) vs ACR (red) as an overall cohort (A) by margin and node status (B‐D). Several patients at risk were censored at time 0 due to immediate post‐operative mortality

Median follow‐up for alive patients was 31 months (i.q.r 20‐48 months). At the time of analysis, there were 2476 deaths in the AC group as compared with 2355 deaths in the ACR group (log rank test *P* < .001). Median OS for ACR group was 22 months (i.qr 13‐43) and that for AC group was 19 months (i.qr 11‐36). Kaplan‐Meier OS estimates for the matched cohort as well as subset populations of R1/N0, R0/N+, and R1/N+ are shown in Figure 2. Only patients in the node positive subgroups (R0/N+ and R1/N+) had improvement in survival from the addition of radiation therapy (log‐rank test *P* < .001 for both). For R1/N0 patients, there was no statistically significant difference in survival (log‐rank test *P* = .633). On univariate Cox proportional hazards model, OS was significantly improved for patients who received ACR over AC (HR 0.84, 95% CI 0.79‐0.88), *P* < .001).

Subset analyses demonstrated OS benefit of ACR compared with AC in N+, R0 patients (HR: 0.82, 95% CI 0.77‐0.88; Median OS 24 vs 20 months, *P* < .001) as well as N+, R1 patients (HR: 0.77, 95% CI 0.68‐0.87; Median OS 17 vs 15 months, *P* < .001); but not in node‐negative, R1 patients (HR: 1.12, 95% CI 0.84‐1.48; Median OS 18 vs 22 months, *P* = .63) (Table 2).

**Table 2:** Association of adjuvant radiation with overall survival of propensity‐score matched patients with high‐risk pancreatic adenocarcinoma

Cohort	Treatment	Median survival (mo)	Hazard ratio (95% CI)	*P*‐value
Overall	Chemotherapy	19	Ref	
	Chemoradiation	22	0.84 (0.79‐0.88)	<.001
R0N+	Chemotherapy	20	Ref	
	Chemoradiation	24	0.82 (0.77‐0.88)	<.001
R+N0	Chemotherapy	22	Ref	
	Chemoradiation	18	1.12 (0.84‐1.48)	.63
R+N+	Chemotherapy	15	Ref	
	Chemoradiation	17	0.77 (0.68‐0.87)	<.001

**Figure 3:**
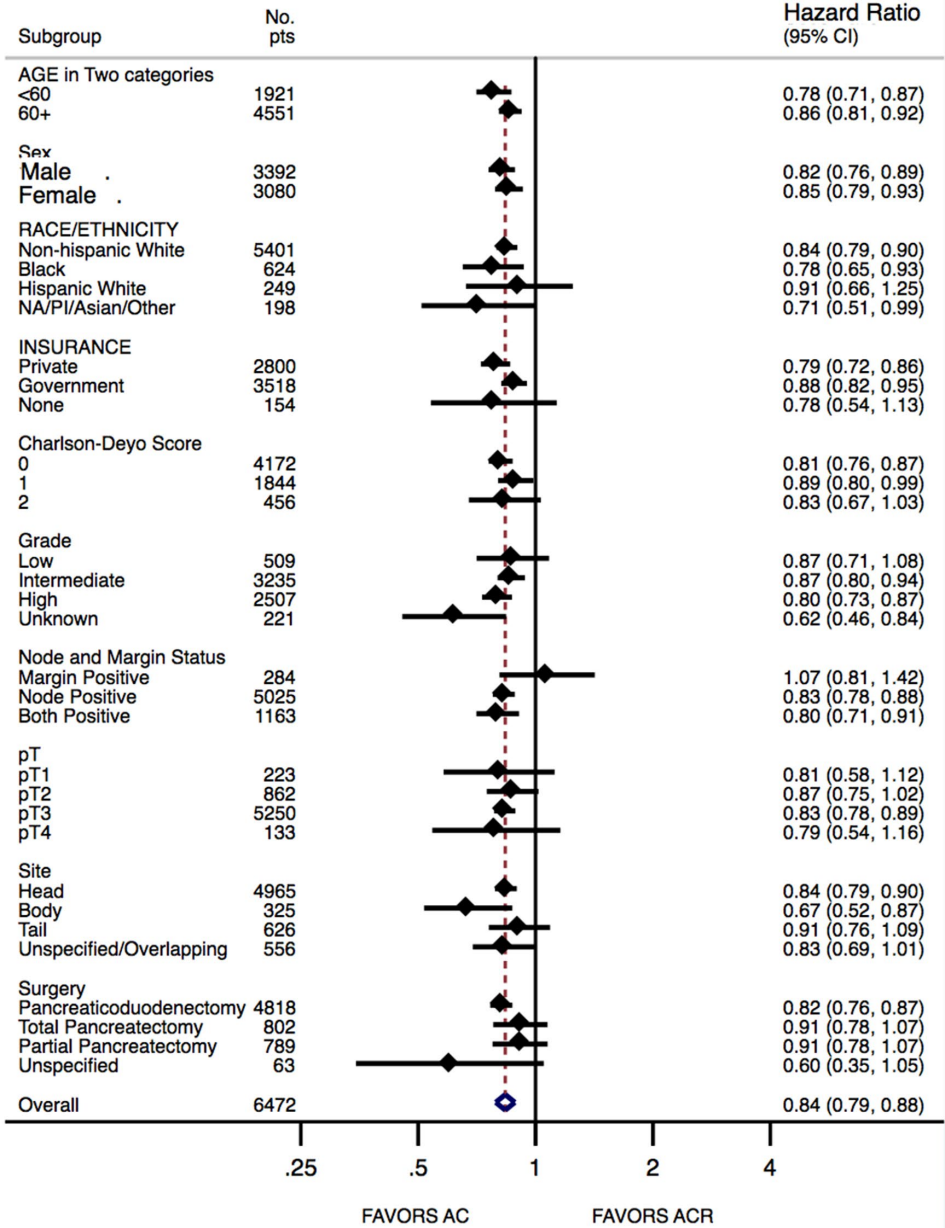
Forest plot for overall survival in the subgroup analysis of propensity score‐matched cohort

A forest plot summarizing which subgroups benefit from the addition of radiation to AC is shown in Figure 3. On this analysis, most subgroups analyzed appear to benefit from ACR. Notably, this analysis failed to show a survival benefit of ACR in R1/N0 subgroup.

### 3.4 Sensitivity analysis

Radiation therapy dose coding in the NCDB can be quite variable as shown by Jacobs et al^27^ To ensure that our results are not impacted by error in coding, we conducted a sensitivity analysis limiting our analysis to clinically acceptable total radiation dose level of 30‐60 Gy. This restricted propensity score‐matched cohort comprised of 7286 patients (3643 in each group).

Groups in the unmatched cohort were significantly imbalanced (Rubin's B = 51.7%, Rubin's R = 0.98; *P* < .001, likelihood ratio test of joint insignificance of all regressors). A propensity‐matching algorithm resulted in two well‐balanced groups, with a bias reduction (Rubin's B = 19.4%, Rubin's R = 1.21; *P* = .07).

The results were similar to the results in non‐sensitivity analysis cohort. On univariate Cox proportional hazards model, OS was significantly improved for patients who received ACR over AC (HR 0.81, 95% CI 0.76‐0.86), *P* < .001). A sub‐group analysis demonstrated that this association was attributable to R0, N+ patients (HR 0.81, 95% CI 0.76‐0.87), *P* < .001); or R1, N+ patients (HR 0.75, 95% CI 0.66‐0.86), *P* < .001). Whereas, the association between survival and addition of radiation to AC was not evident for R1, N0 patients (HR 0.94, 95% CI 0.70‐1.26), *P* < .667).

## 4 DISCUSSION

Pancreatic cancer is now widely recognized as a systemic disease regardless of the clinical stage at presentation.^28^ Despite this recognition, local control with complete surgical resection, when possible, is the mainstay of curative‐intent treatment.^29^, ^30^ Following curative‐intent surgery, adjuvant systemic therapy to treat occult metastatic disease is the standard‐of‐care.^5^ Most patients despite curative‐intent resection and adjuvant systemic therapy will recur. Of the patients that recur 50%‐85% will have a locoregional component^2^, ^3^, ^4^ and ~25% will have a locoregional recurrence without distant failure, suggesting the need for improved local control.^4^ However, enhancing further local control with the addition of radiation therapy remains controversial.

To that end, this is a large study of a contemporary cohort of patients treated in various hospitals across the United States. In this study, we aimed to determine the benefit of adjuvant radiation in patients known to be at high‐risk of local failure, that is, those with positive lymph nodes and/or microscopically positive margins. The results of this well‐balanced propensity score‐matched analysis demonstrate a modest 3‐month improvement in median OS with the addition of radiation to AC. When we analyzed well‐matched subgroups, this survival benefit was noted in all node‐positive patients regardless of margin status. However, the study failed to show this survival benefit for margin‐positive, node‐negative patients.

The benefit of adjuvant chemoradiation therapy was first demonstrated in the GITSG trial that randomized patients to surgery alone or adjuvant 5‐FU chemoradiation followed by maintenance 5‐FU.^7^ Even though this was a small study, it demonstrated superiority of adjuvant chemoradiation over surgery alone. To reproduce these findings EORTC conducted a larger trial with a similar design.^8^ This time, the two treatment arms were not statistically different. To seek clarity on the benefit of adjuvant therapy in pancreatic cancer patients, a large Phase III randomized trial with a 2x2 factorial design was conducted in Europe.^9^ The treatment arms comprised observation, AC alone, adjuvant radiation alone, and adjuvant chemoradiation. This trial demonstrated a survival benefit with AC but found that adjuvant radiation was detrimental to OS. Despite concerns of quality control with radiation and trial design, the results impacted clinical practice widely. A recent phase III trial of adjuvant chemoradioimmunotherapy (5‐FU, Cisplatin, Interferon alpha‐2b) vs chemotherapy (5‐FU) also failed to show any significant improvement in survival.^31^ An ongoing Phase III trial (Radiation therapy oncology group—RTOG 0848) is evaluating the role of radiation therapy in patients who do not progress after first 5 cycles of chemotherapy.^32^ At present adjuvant radiation is used selectively with most institutions. American Society of Clinical Oncology clinical practice guidelines recommend administration of radiation following AC in microscopic margin‐positive and node‐positive pancreatic cancers.^12^, ^13^

This study differs from prior retrospective studies in that it uses a contemporary cohort of patients.^14^, ^17^ This is important because radiation treatment and planning technology has significantly improved at the turn of the century resulting in decreased toxicity. In addition, contrary to prior studies,^10^, ^11^, ^14^, ^15^, ^16^, ^17^, ^30^ this study specifically focuses on the high‐risk group of patients that are most likely to get adjuvant radiation in the scope of current clinical practice. This makes these results clinically relevant. One recent study evaluated the role of ACR in patients with R1 pancreatic resections.^10^ On multivariable analysis ACR was superior to AC. However, this study excluded node positive patients with R0 resection. Furthermore, the cases and controls were unmatched. Consistent with prior studies, the present study demonstrates a survival benefit for adding radiation to AC. However, we found that this benefit is largely attributable to the treatment of nodal disease. Specifically, despite a large sample size, the present study failed to show an OS benefit of adding radiation to AC in node‐negative patients with microscopically positive margins. This observation agrees with a large multi‐institutional study spanning the same period.^33^ Furthermore, by performing propensity score matching, we attempt to minimize the treatment selection bias that have limited the interpretation from other retrospective studies.

The interpretation of these results is limited because of a retrospective design. Currently, the NCDB does not record data on specific type of chemotherapy, treatment failure, patterns of treatment failure and local‐recurrence free survival, and these variables could not therefore be assessed. For pancreatic cancer, these endpoints would not change the conclusion of the study but may provide more insight. To minimize bias due to treatment selection, propensity score matching was performed. While propensity score matching algorithms allow balancing of known covariates, unknown confounders can only be controlled in a randomized trial such as the RTOG 0848. Additional factors that are used in treatment selection at times and not reliably recorded in the NCDB include perineural invasion, lymphovascular invasion, cancer antigen 19‐9 levels. However, the study accounted for the major factors used in the selection of adjuvant therapy. Over the last decade, combination multi‐agent chemotherapy (eg, FOLFIRINOX and gemcitabine with nab‐paclitaxel) is being increasingly used for adjuvant treatment of patients in good performance status with limited comorbidities. Because we do not have data on specific chemotherapy used in this cohort, we advise caution in extrapolating the findings to patients who receive contemporary chemotherapy regimens.

In summary, addition of radiation to AC was associated with a clinically small but meaningful increase in survival of patients undergoing curative‐intent pancreatic resections. This association was not evident in patients with microscopically positive margins but node‐negative disease and larger studies will be needed. Stratified analysis of RTOG 0848 trial will provide further clarity regarding the use of radiation in this subgroup of patients. In the absence of data from well‐designed high‐quality randomized trials, this study can inform treatment planning decisions in multidisciplinary tumor boards.

## CONFLICT OF INTEREST

Nothing to disclose.

## AUTHOR CONTRIBUTIONS

MR and YF contributed to conception and design. MR and LGM contributed to provision of study materials or patients. MR and AMB contributed to collection and assembly of data. MR and AMB contributed to manuscript writing. All authors contributed to data analysis and interpretation, final approval of manuscript, and all authors are accountable for all aspects of the work.
