# Supplementary material for: Adjuvant chemotherapy versus chemoradiation in high‐risk pancreatic adenocarcinoma: A propensity score‐matched analysis
Source: Cancer Med. 2019 Aug 15;8(13):5881–90. doi: 10.1002/cam4.2491 (PMC6792522; doi:10.1002/cam4.2491)
Supplement: Supplementary file 1 [file CAM4-8-5881-s001.docx]

**Figure S1**. Kernel Density Plot of Propensity Scores before matching (unmatched) and after matching (matched) patients stratified by the two comparison groups. (Adjuvant Chemoradiation vs. Adjuvant Chemotherapy)
